# Insulin Requirement and Complications Associated With Serum C-Peptide Decline in Patients With Type 1 Diabetes Mellitus During 15 Years After Diagnosis

**DOI:** 10.3389/fendo.2022.869204

**Published:** 2022-04-19

**Authors:** Junghwan Suh, Hae In Lee, Myeongseob Lee, Kyungchul Song, Han Saem Choi, Ahreum Kwon, Ho-Seong Kim, Hyun Wook Chae

**Affiliations:** Department of Pediatrics, Severance Children’s Hospital, Endocrine Research Institute, College of Medicine, Yonsei University, Seoul, South Korea

**Keywords:** type 1 diabetes mellitus, C-peptide, diabetes complications, insulin, pancreatic beta cell function

## Abstract

**Objective:**

C-peptide is conventionally used in assessing pancreatic function in patients with diabetes mellitus. The clinical significance of this molecule during the course of type 1 diabetes mellitus (T1DM) has been recently revisited. This study aimed to investigate the natural course of C-peptide in T1DM patients over the period of 15 years and analyze the association between the residual C-peptide and diabetes complications.

**Methods:**

This retrospective study included a total of 234 children and adolescents with T1DM. Patient data including sex, age at diagnosis, anthropometric measures, daily insulin dose, serum HbA1c, post-prandial serum C-peptide levels, lipid profiles, and diabetic complications at the time of diagnosis and 1, 3, 5, 10, and 15 years after diagnosis were retrospectively collected.

**Results:**

Among the 234 patients, 101 were men and 133 were women, and the mean patient age at initial diagnosis was 8.3 years. Serum C-peptide decreased constantly since the initial diagnosis, and showed a significant decline at 3 years after diagnosis. At 15 years after diagnosis, only 26.2% of patients had detectable serum C-peptide levels. The subgroup with older patients and patients with higher BMI standard deviation score showed higher mean serum C-peptide, but the group-by-time results were not significant, respectively. Patients with higher serum C-peptide required lower doses of insulin and had fewer events of diabetic ketoacidosis.

**Conclusion:**

Serum C-peptide decreased consistently since diagnosis of T1DM, showing a significant decline after 3 years. Patients with residual C-peptide required a lower dose of insulin and had a lower risk for diabetic ketoacidosis.

## Introduction

Type 1 diabetes mellitus (T1DM) is an autoimmune disease, which is characterized by decreased insulin secretion from the pancreas. As insulin has a short half-life and variable first-pass hepatic extraction, it is inaccurate to utilize insulin for assessing pancreatic beta cell function (1). C-peptide, a polypeptide that connects insulin’s A-chain to B-chain, is co-secreted with insulin during the enzymatic cleavage of proinsulin to insulin (2). C-peptide is more stable, and have longer half-life than insulin, which is why it is widely used for the evaluation of insulin secretion capacity in patients with T1DM.

Previously, C-peptide was considered as a byproduct generated during insulin synthesis and only used in estimating the pancreatic function of diabetes mellitus (DM) patients. However, many recent studies have re-evaluated the clinical importance of C-peptide as a representative marker of insulin secretary function. Some studies have reported that residual beta cell function displays a protective effect against the development of diabetes complications and hypoglycemia (3, 4). Moreover, some studies explored the potential of C-peptide in therapeutics such as C-peptide replacement therapy (5–7). Not only that, but research on the genetic basis of residual beta cell function and its association with specific gene variants has attracted the significant attention recently (8, 9). Furthermore, several studies have confirmed the trend of declining C-peptide levels over the course of T1DM (1, 10). However, longitudinal studies are scarce, and the results are not consistent. This highlights the need for more clinical data on the changes of serum C-peptide levels in patients with T1DM and their correlation with residual beta cell function and patients’ prognosis.

In this study, we longitudinally investigated the natural course of serum C-peptide in T1DM patients over a 15-year duration, and evaluated the factors which might be associated with serum C-peptide decline. In addition, clinical characteristics and development of diabetes complications are compared between the group with preserved C-peptide production and those with depleted beta cells.

## Materials and Methods

### Patients

We retrospectively reviewed T1DM patients who visited Severance Children’s Hospital between March 2017 and December 2020. Diagnosis of T1DM was made following the diagnostic criteria for DM at the initial visit (11), by fulfilling one or more of the following criteria: positive autoantibodies (islet cell antibody, anti-glutamic acid decarboxylase antibody, and insulin autoantibody), initial diabetic ketoacidosis (DKA) events, and fasting serum C-peptide level lower than 0.6 ng/mL. Patients with a disease duration of over 15 years with the presence of serial C-peptide with 3 or more measurements during the follow-up period were included, and patients with insufficient data, less than 15 years of follow-up period, and misdiagnosed patients were excluded.

This study was approved by the institutional review board of Severance Hospital, Yonsei University College of Medicine in Seoul, Korea (No. 3-2017-0119). The requirement to obtain informed consent was waived due to the retrospective nature of the study.

### Study Design

Patient data including sex, age at diagnosis, duration of disease, family history of DM, anthropometric measures, daily insulin dose, and diabetes complications were retrospectively collected at the time of diagnosis and 1, 3, 5, 10, and 15 years after diagnosis. Also, laboratory findings including post-prandial serum C-peptide, Glycated hemoglobin A (HbA1c), and lipid profiles were collected at the same time points.

### Assessment of Clinical Parameters and Diabetes Complications

Family history included all types of DM in any relative up to the third-degree. Height standard deviation score (SDS), weight SDS, and body mass index (BMI) SDS were calculated using the standard growth charts for Korean children and adolescents (12). Serum C-peptide levels were measured using the radioimmunoassay technique (Daiichi, Tokyo, Japan), and serum HbA1c was assessed by high-performance liquid chromatography (Variant II, Variant Turbo, Bio-rad, Hercules, CA, USA). Lipid profiles were evaluated by a non-fasting lipid screening test, including total cholesterol, triglyceride, low-density lipoprotein, and high-density lipoprotein. Total cholesterol and triglyceride were determined by an enzymatic method (Sekisui Medical Co., Tokyo, Japan), and low-density lipoprotein and high-density lipoprotein were measured by homogenous direct assay (Sekisui Medical Co., Tokyo, Japan).

Positive DKA history was recorded when a patient had one or more DKA events according to diagnostic criteria (13), with DKA events at initial diagnosis being excluded. Annual screening for diabetic complications has been performed starting from age 11 years with 5 years of diabetes durations. Peripheral neuropathy was confirmed by a neurologist with nerve conduction studies using electromyography. Motor and sensory nerve conduction velocities were measured in the median, ulnar, tibial, sural, and common peroneal nerves following standard methods. Nephropathy was confirmed when albumin excretion in a 24-hour urine collection was more than 30 mg in repeated tests. Diabetic retinopathy was diagnosed by an ophthalmologist with dilated fundus examination.

### Statistical Analysis

Statistical analyses were performed using SAS version 9.4 (SAS Institute Inc., Cary, NC, USA). Linear mixed model was applied to compare clinical and biochemical characteristics over 15 years and analyze the factors associated with serum C-peptide levels. The decline of serum C-peptide levels was described using the mean profile graph and linear mixed model. Independent two-sample t-test was used to compare parameters between the two groups with low and high serum C-peptide levels. Chi-square test and Fisher’s exact test were used to assess the association between serum C-peptide level and diabetes complications. p < 0.05 was considered statistically significant.

## Results

### Characteristics of T1DM Patients

**Figure 1:**
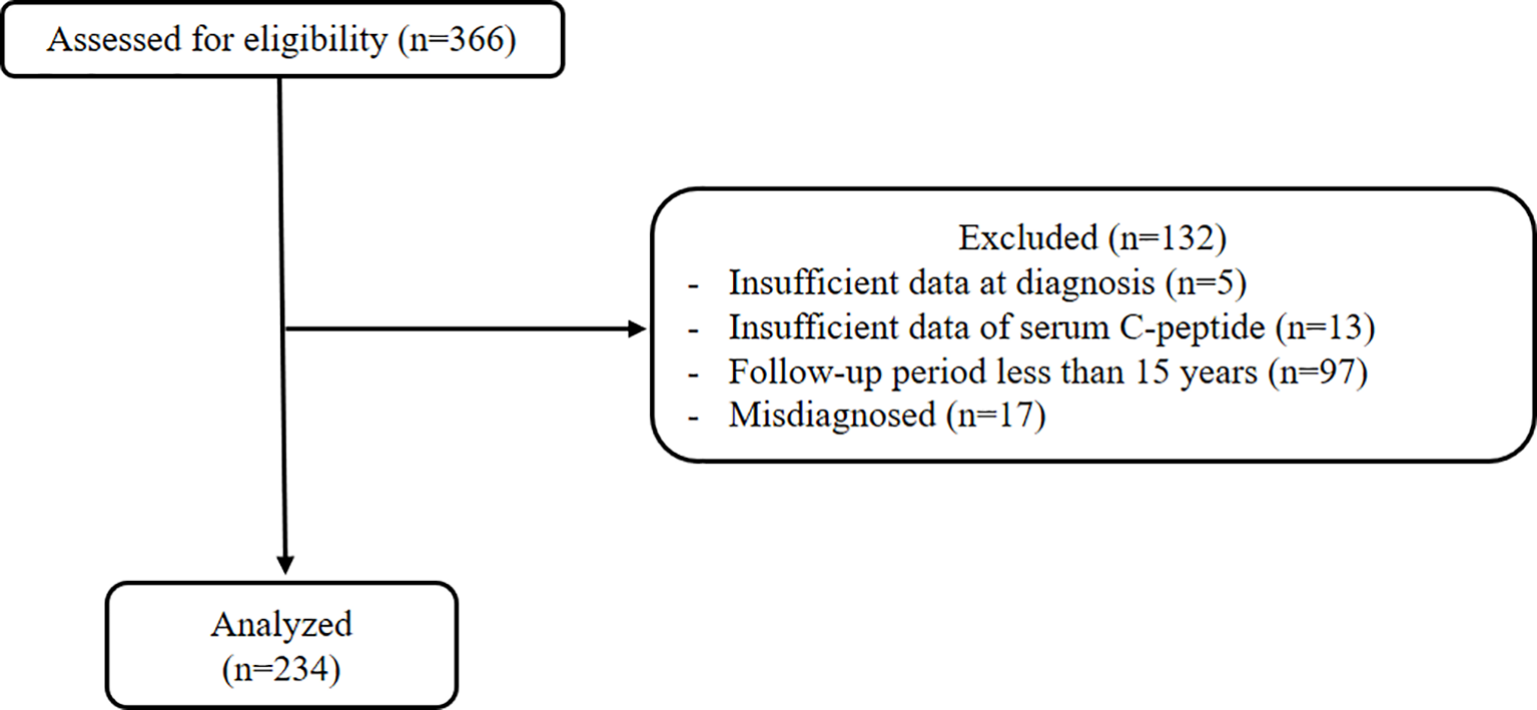
Flow chart for patient selection.

Among 366 T1DM patients, 17 patients who were initially misdiagnosed with T1DM were excluded. Also, a total of 132 patients were excluded for not fulfilling the eligibility criteria, and data on the remaining 234 patients were included in the final analysis (**Figure 1**). All patients were under intensive insulin therapy by multiple daily injections or through an insulin pump since 2010.

Of the total 234 T1DM patients, 101 were male and 133 were female. The mean patient age at initial diagnosis was 8.3 years. Mean serum C-peptide level at diagnosis was 0.85 ng/mL, and mean HbA1c was 11.95%. Height SDS significantly decreased during the 15-year follow-up period, while weight SDS and BMI SDS had increased (overall p value <0.001). Clinical and biochemical characteristics of T1DM patients are presented in **Table 1**. Also, presence of diabetes complications at each time point are presented in **Supplementary Table 1**.

**Table 1:** Clinical and biochemical characteristics of type 1 diabetes mellitus patients over 15 years.

	Baseline	Year 1	Year 3	Year 5	Year 10	Year 15
Number of patients, n	234	221	223	222	215	183
Age, years	8.3 ± 3.8	9.2 ± 3.7	11.2 ± 3.6	13.4 ± 3.7	18.1 ± 3.8	22.9 ± 3.6
C-peptide, ng/mL	0.85 ± 1.00	0.78 ± 0.61	0.63 ± 0.76*	0.46 ± 0.73*	0.37 ± 0.84*	0.20 ± 0.64*
HbA1c, %	11.95 ± 4.04	8.59 ± 3.10*	9.08 ± 2.65*	9.03 ± 2.55*	9.01 ± 2.44*	8.52 ± 1.95*
Height SDS	0.52 ± 1.24	0.47 ± 1.24	0.35 ± 1.17*	0.29 ± 1.23*	0.07 ± 1.17*	-0.07 ± 1.18*
Weight SDS	-0.08 ± 1.18	0.23 ± 1.02*	0.20 ± 0.97*	0.23 ± 0.96*	0.15 ± 0.95*	0.21 ± 1.21*
BMI SDS	-0.55 ± 1.31	-0.03 ± 0.91*	0.05 ± 0.83*	0.10 ± 0.84*	0.11 ± 0.87*	0.25 ± 1.15*
Total cholesterol, mg/dL	185.7 ± 62.2	160.9 ± 28.8*	170.5 ± 37.2*	171.2 ± 37.3*	184.1 ± 73.4	179.5 ± 37.6
Triglyceride, mg/dL	183.5 ± 298.3	85.7 ± 58.0*	90.8 ± 70.9*	95.4 ± 89.9*	98.8 ± 83.7*	91.0 ± 71.5*
HDL-Cholesterol, mg/dL	48.7 ± 17.3	54.5 ± 16.8*	56.6 ± 15.8*	55.9 ± 16.0*	60.8 ± 16.2*	60.0 ± 14.4*
LDL-Cholesterol, mg/dL	102.5 ± 43.9	90.8 ± 29.6*	94.2 ± 30.7	98.2 ± 33.1	105.2 ± 34.6	104.0 ± 29.8
Daily insulin dose, units/kg/day	0.77 ± 0.40	0.75 ± 0.32	0.91 ± 0.36*	0.96 ± 0.38*	0.89 ± 0.34*	0.85 ± 0.32*

Data are presented as mean ± SD.

*p < 0.05, compared to baseline values.

HbA1c, glycated hemoglobin A; SDS, standard deviation score; BMI, body mass index; HDL, high-density lipoprotein; LDL, low-density lipoprotein; SD, standard deviation.

### Decline of Serum C-Peptide Levels

**Figure 2:**
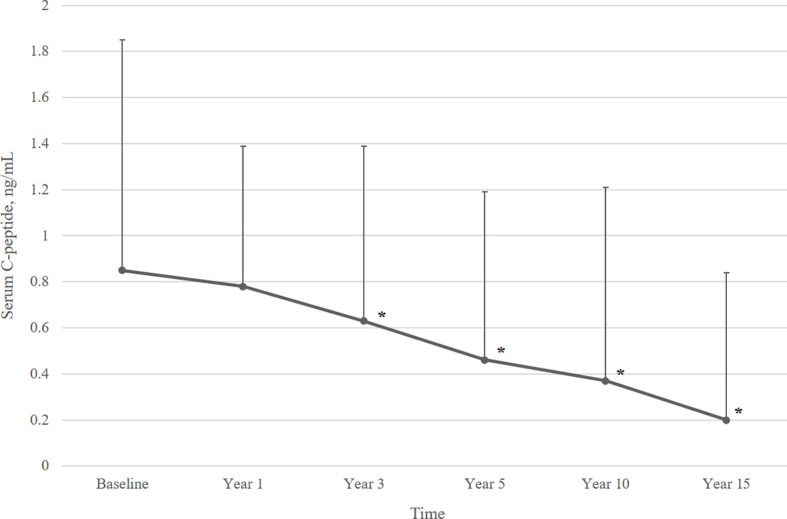
Mean serum C-peptide levels over 15 years of follow-up. Data are presented as mean ± SD. Overall p value <0.001. *p<0.05, compared to baseline serum C-peptide level.

**Figure 3:**
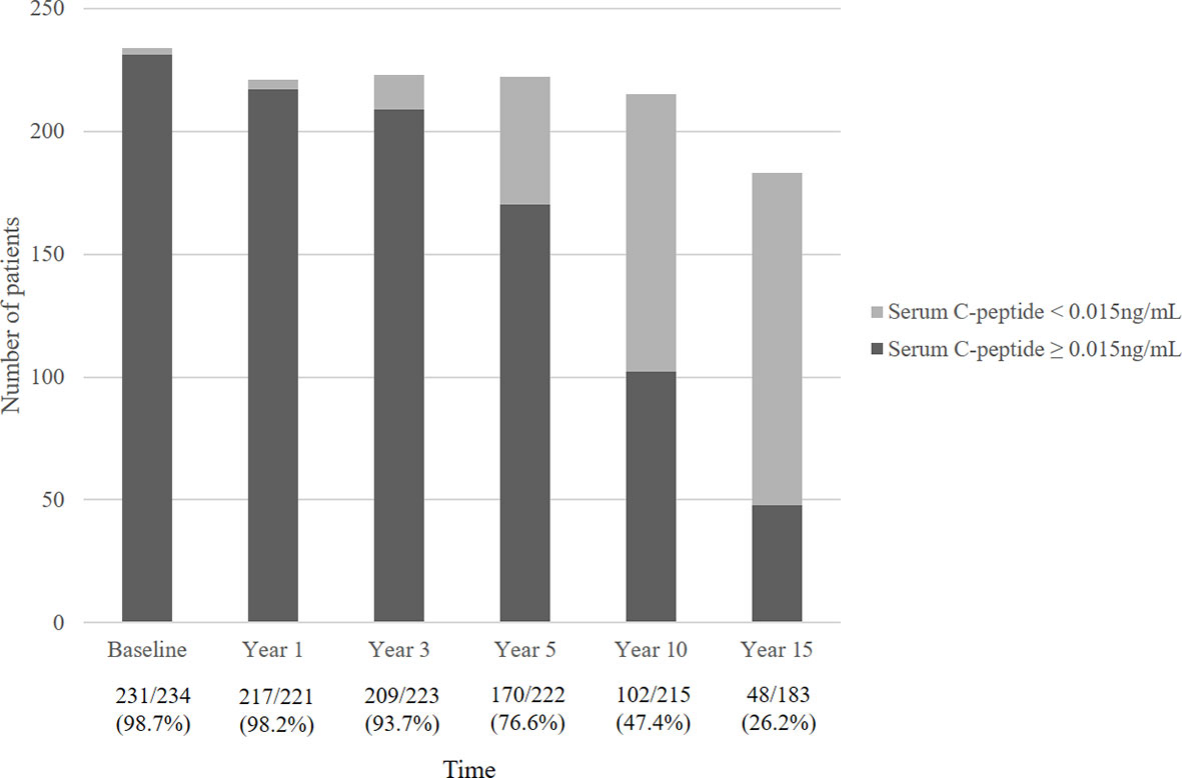
The proportion of patients with residual serum C-peptide secretion.

**Figure 2** describes the mean serum C-peptide level during the 15-year follow-up period. Serum C-peptide levels decreased constantly following initial diagnosis (overall p<0.001) and showed a significant decline at 3 years after diagnosis. Also, the proportion of patients with detectable serum C-peptide levels decreased over the disease period. Over ninety percent of T1DM patients had serum C-peptide over 0.015 ng/mL until 3 years after diagnosis, which decreased to 76.6% at year 5, and 47.4% at year 10. Only 26.2% of patients had detectable serum C-peptide levels (≥0.015 ng/mL) at year 15 (**Figure 3**).

### Factors Associated With Serum C-Peptide

Stratified analysis was performed to assess factors that correlate with serum C-peptide levels (**Table 2**). Mean serum C-peptide levels were higher in patients with older age at diagnosis (p<0.001). However, the group-by-time results were not statistically significant when serial measurement for 15 years was taken into consideration (overall p=0.061). Also, the group of patients with higher BMI scores at diagnosis showed higher mean serum C-peptide levels (p<0.001), but the group-by-time results were not significant (overall p=0.726). There were no remarkable differences between groups stratified by sex, family history of DM, and history of diabetes complications.

**Table 2:** Factors associated with serum C-peptide.

		Serum C-peptide, ng/mL						Overall p value
		Baseline	Year 1	Year 3	Year 5	Year 10	Year 15	
Sex	Male (n=101)	0.91 ± 1.20	0.76 ± 0.64	0.69 ± 1.01	0.51 ± 0.89	0.42 ± 0.93	0.21 ± 0.76	0.518
	Female (n=133)	0.81 ± 0.81	0.85 ± 0.70	0.66 ± 0.68	0.47 ± 0.64	0.35 ± 0.78	0.22 ± 0.56	
Family history of DM	Yes (n=68)	1.03 ± 1.38	0.91 ± 0.85	0.86 ± 1.28	0.51 ± 0.66	0.43 ± 0.77	0.32 ± 0.69	0.301
	No (n=166)	0.78 ± 0.79	0.77 ± 0.57	0.59 ± 0.55	0.48 ± 0.80	0.36 ± 0.88	0.17 ± 0.63	
Age at diagnosis, years	≤ 8.3 (n=117)	0.59 ± 0.32	0.67 ± 0.52	0.60 ± 0.70	0.38 ± 0.36	0.25 ± 0.61	0.07 ± 0.25	0.061
	> 8.3 (n=117)	1.12 ± 1.33	0.95 ± 0.77	0.74 ± 0.96	0.58 ± 0.99	0.52 ± 1.03	0.39 ± 0.91	
BMI SDS at baseline	< 0 (n=132)	0.73 ± 0.92	0.62 ± 0.33	0.46 ± 0.33	0.32 ± 0.29	0.24 ± 0.59	0.08 ± 0.28	0.726
	≥ 0 (n=63)	1.06 ± 1.04	1.03 ± 0.90	1.00 ± 1.34	0.69 ± 1.12	0.61 ± 1.12	0.38 ± 0.96	
Diabetes complications	None (n=106)	0.87 ± 1.15	0.76 ± 0.64	0.58 ± 0.67	0.39 ± 0.58	0.37 ± 0.87	0.17 ± 0.52	0.141
	1 or more (n=128)	0.83 ± 0.81	0.87 ± 0.70	0.77 ± 0.98	0.59 ± 0.90	0.39 ± 0.83	0.25 ± 0.74	

Data are presented as mean ± SD.

DM, diabetes mellitus; BMI, body mass index; SDS, standard deviation score; SD, standard deviation.

### Comparison Between Two Groups Divided by Serum C-Peptide Level

We divided patients into two groups according to serum C-peptide level at baseline and after 15 years, and we compared laboratory findings and prevalence of diabetes complications at year 15 between the two groups (**Table 3**). Results showed that patients with higher levels of serum C-peptide at diagnosis and 15 years after diagnosis had been treated with lower doses of insulin. There were no significant differences in serum HbA1c and lipid profiles between the two groups. In subgroup analysis divided by serum C-peptide level at 15 years after diagnosis, DKA events occurred more frequently in the group with lower serum C-peptide levels than group with preserved C-peptide levels (23.7% vs. 6.8%, p=0.014).

**Table 3:** Comparison between two groups divided by serum C-peptide level at diagnosis and 15 years after diagnosis.

	Baseline			Year 15		
	C-peptide < 0.5 ng/mL(n=77)	C-peptide ≥ 0.5 ng/mL(n=157)	p value	C-peptide < 0.015 ng/mL(n=135)	C-peptide ≥ 0.015 ng/mL(n=48)	p value
Laboratory findings						
HbA1c, %	9.67 ± 1.76	9.37 ± 1.90	0.245	9.69 ± 1.84	9.69 ± 2.15	0.990
Total cholesterol, mg/dL	174.8 ± 29.2	175.5 ± 30.5	0.870	175.4 ± 28.8	182.5 ± 32.7	0.160
Triglyceride, mg/dL	116.0 ± 82.0	100.9 ± 58.4	0.149	104.0 ± 62.3	129.7 ± 102.5	0.108
HDL-Cholesterol, mg/dL	56.4 ± 13.9	55.2 ± 12.8	0.530	56.4 ± 13.1	52.0 ± 15.7	0.064
LDL-Cholesterol, mg/dL	97.0 ± 25.3	101.4 ± 24.7	0.209	99.8 ± 22.3	107.5 ± 30.8	0.116
Daily insulin dose, units/kg/day	0.90 ± 0.26	0.76 ± 0.28	< 0.001	0.88 ± 0.21	0.74 ± 0.37	0.034
Diabetes complications						
Diabetic retinopathy	17 (22.1%)	43 (27.4%)	0.382	35 (25.9%)	14 (31.8%)	0.447
Peripheral neuropathy	17 (22.1%)	37 (23.6%)	0.799	34 (25.2%)	10 (22.7%)	0.742
Diabetic nephropathy	16 (20.8%)	36 (22.9%)	0.710	28 (20.7%)	10 (22.7%)	0.780
Diabetic ketoacidosis	16 (20.8%)	23 (14.6%)	0.237	32 (23.7%)	3 (6.8%)	0.014

Data are presented as mean ± SD or number of patients (%).

HbA1c, glycated hemoglobin A; HDL, high-density lipoprotein; LDL, low-density lipoprotein; SD, standard deviation.

## Discussion

In this retrospective study, we longitudinally tracked 234 T1DM patients for a period of 15 years and demonstrated a natural decline of serum C-peptide levels. Serum C-peptide significantly decreased at 3 years after diagnosis, while 26.2% of patients still had maintained detectable levels of serum C-peptide at 15 years after diagnosis. Also, as patients with residual pancreatic beta cell function required lower doses of insulin and showed a lower incidence of DKA events, regular follow-up of serum C-peptide could be considered in longitudinal management of type 1 diabetes patients.

Serum C-peptide had been used in assessing pancreatic function in DM patients for a long time. Recently, several studies started investigating the clinical effect of residual beta cell function in T1DM patients and tried to elucidate the natural course of C-peptide levels in these patients. A number of previous studies reported that the insulin secreting capacity becomes depleted in T1DM patients soon after diagnosis (14, 15), but results from the Joslin Medalist Study showed persistent insulin production in 67.4% of participants at 50 years after diagnosis (16). Also, several recent studies presented a slow decline of serum C-peptide levels and preservation of the insulin secreting capacity (17–19). Previously, a preliminary study conducted with a small number of T1DM patients and showed a slight increase in serum C-peptide levels after diagnosis followed by significant decrease 5 years later (20). However, the results of this study demonstrate a continuous decline in serum C-peptide levels following diagnosis and an especially significant decline from baseline after 3 years. Also, 26.2% of patients had residual C-peptide secretion at 15 years after diagnosis, which is similar to the results of recent studies that reported long-term preservation of pancreatic beta cell function.

Several hypotheses are presented to explain the clinical implications of residual C-peptide secretion in T1DM. Some previous studies suggested that C-peptide acts as an endogenous antioxidant, which protects pancreatic beta cells by increasing catalase expression and reducing peroxisomal oxidative stress (21–23). Additionally, Thivolet et al. reported an association between residual C-peptide levels and reduction in response to glucagon-like peptide-1 (GLP-1) (24). Although the mechanism is not clearly elucidated yet, the clinical benefit from preserved C-peptide secretion in DM patients is widely known. Microvascular complications such as diabetic retinopathy and nephropathy were found to be less likely to develop in patients with residual C-peptide production (4, 17, 19, 25, 26), but the results were not always consistent among studies (27, 28). Also, preserved beta cell function is reported to be related to a decreased risk of hypoglycemia and decreased insulin requirement (25–27, 29). Moreover, with the wide use of continuous glucose monitoring (CGM) systems, recent studies reported on the importance of glycemic variability and its association with C-peptide levels. Patients with residual C-peptide production had a lower mean blood glucose level and higher time in range (30), and fasting C-peptide levels were negatively correlated with glucose coefficient of variation by CGM (31). The present study confirmed the lower insulin requirement and lower incidence of DKA events in patients with residual C-peptide secretion, but no significant association with HbA1c levels and microvascular complications. Additional analyses including hypoglycemia and glucose variability using the ambulatory glucose profile report from CGM, can be considered in future studies.

Preserved beta cell function in T1DM has recently received increased attention from the research community for its various metabolic benefits and significant effect on clinical outcomes. This is why several studies were performed to assess the factors that are associated with residual C-peptide levels. Older age at diagnosis is one of the most commonly discussed factors that are associated with residual beta cell function (17, 32–34). C-peptide level at initial diagnosis is lower in prepubertal children than in adolescent and young adults and declines more rapidly in younger children (35). The results of the present study showed higher mean serum C-peptide level in the older age group than younger group, but the results were not statistically significant in the group-by-time model. Also, higher BMI was proven to be another factor related to residual C-peptide production (36, 37), but some studies reported variation in this relationship among age groups (38). Our data showed no significant correlation between BMI SDS and serum C-peptide levels when taking the duration since diagnosis into consideration, but the mean C-peptide level was higher in the group with a BMI SDS over zero. Furthermore, intensive insulin therapy is known to sustain endogenous insulin secretion, lower the risk for hypoglycemia, and reduce diabetes complications in T1DM patients (34, 39). All patients in our study were treated with intensive insulin therapy by multiple daily injections or through an insulin pump since 2010.

There are some limitations to this study. We excluded a number of patients who were initially misdiagnosed as T1DM, which turned out to be type 2 DM or monogenic diabetes. However, other misdiagnosed patients with equivocal clinical and laboratory findings might have remained. Further workups, including genetic screening for monogenic diabetes, would be helpful in differentiating T1DM patients from patients with other types of DM. Also, as it is difficult to routinely check both fasting and post-prandial serum C-peptide levels during follow-up visits in the outpatient setting, so we only examined meal stimulated post-prandial C-peptide levels. More detailed study outcomes would have been possible if we also checked fasting serum C-peptide levels. In addition, as the patients were diagnosed with T1DM at a mean age of 8.3 years, the majority of the patients were in their young adulthood at the time of study analysis. The prevalence of microvascular complications would be relatively low in this age group. Lastly, this study was performed in a single tertiary center with a retrospective design. Further studies with larger sample sizes and longer follow-up periods would provide more accurate results. Nevertheless, our study has a significant strength owing to the relatively large number of pediatric T1DM patients with 15 years of follow-up.

## Conclusions

Serum C-peptide decreased consistently following diagnosis of T1DM, reaching a significant change at 3 years after diagnosis. Patients with residual beta cell function required lower doses of insulin and had a lower risk for developing DKA.

## Data Availability Statement

The raw data supporting the conclusions of this article will be made available by the authors, without undue reservation.

## Ethics Statement

The studies involving human participants were reviewed and approved by Severance Hospital, Yonsei University College of Medicine. Written informed consent for participation was not provided by the participants’ legal guardians/next of kin because: The requirement to obtain informed consent was waived due to the retrospective nature of the study.

## Author Contributions

JS and HWC conceptualized and designed the study, drafted the initial manuscript, and reviewed and revised the manuscript. HIL, ML, KS, and HSC assisted on the study design, collected data and conducted data analyses. AK and HK reviewed, suggested subanalyses, and revised the manuscript. All authors contributed to the article and approved the submitted version.

## Conflict of Interest

The authors declare that the research was conducted in the absence of any commercial or financial relationships that could be construed as a potential conflict of interest.

## Publisher’s Note

All claims expressed in this article are solely those of the authors and do not necessarily represent those of their affiliated organizations, or those of the publisher, the editors and the reviewers. Any product that may be evaluated in this article, or claim that may be made by its manufacturer, is not guaranteed or endorsed by the publisher.

## Supplementary Material

Supplementary Table 1 Presence of diabetes complications at each time point. Data are presented as number of patients (%). Diabetic ketoacidosis event at initial diagnosis is excluded.

## References

[1] Sherry NA Tsai EB Herold KC. Natural History of Beta-Cell Function in Type 1 Diabetes. Diabetes (2005) 54 Suppl 2:S32–9. doi: 10.2337/diabetes.54.suppl_2.s32 16306337

[2] Palmer JP Fleming GA Greenbaum CJ Herold KC Jansa LD Kolb H. C-Peptide Is the Appropriate Outcome Measure for Type 1 Diabetes Clinical Trials to Preserve Beta-Cell Function: Report of an Ada Workshop, 21-22 October 2001. Diabetes (2004) 53(1):250–64. doi: 10.2337/diabetes.53.1.250 14693724

[3] Vantyghem MC Raverdy V Balavoine AS Defrance F Caiazzo R Arnalsteen L. Continuous Glucose Monitoring After Islet Transplantation in Type 1 Diabetes: An Excellent Graft Function (B-Score Greater Than 7) Is Required to Abrogate Hyperglycemia, Whereas a Minimal Function Is Necessary to Suppress Severe Hypoglycemia (B-Score Greater Than 3). J Clin Endocrinol Metab (2012) 97(11):E2078–83. doi: 10.1210/jc.2012-2115 PMC3485599 22996144

[4] Steffes MW Sibley S Jackson M Thomas W. Beta-Cell Function and the Development of Diabetes-Related Complications in the Diabetes Control and Complications Trial. Diabetes Care (2003) 26(3):832–6. doi: 10.2337/diacare.26.3.832 12610045

[5] Wahren J Kallas A Sima AA. The Clinical Potential of C-Peptide Replacement in Type 1 Diabetes. Diabetes (2012) 61(4):761–72. doi: 10.2337/db11-1423 PMC3314360 22442295

[6] Washburn RL Mueller K Kaur G Moreno T Moustaid-Moussa N Ramalingam L. C-Peptide as a Therapy for Type 1 Diabetes Mellitus. Biomedicines (2021) 9(3):270. doi: 10.3390/biomedicines9030270 33800470 PMC8000702

[7] Bhatt MP Lim YC Ha KS. C-Peptide Replacement Therapy as an Emerging Strategy for Preventing Diabetic Vasculopathy. Cardiovasc Res (2014) 104(2):234–44. doi: 10.1093/cvr/cvu211 25239825

[8] Yu MG Keenan HA Shah HS Frodsham SG Pober D He Z. Residual B Cell Function and Monogenic Variants in Long-Duration Type 1 Diabetes Patients. J Clin Invest (2019) 129(8):3252–63. doi: 10.1172/jci127397 PMC6668678 31264968

[9] Feng Y Zhang Y Chen Y Chen S Shen M Fu Q. The Associations Between Three Genome-Wide Risk Variants for Serum C-Peptide of T1d and Autoantibody-Positive T1d Risk, and Clinical Characteristics in Chinese Population. J Hum Genet (2020) 65(3):297–303. doi: 10.1038/s10038-019-0705-2 31827251

[10] Snorgaard O Lassen LH Binder C. Homogeneity in Pattern of Decline of Beta-Cell Function in Iddm. Prospective Study of 204 Consecutive Cases Followed for 7.4 Yr. Diabetes Care (1992) 15(8):1009–13. doi: 10.2337/diacare.15.8.1009 1505301

[11] Mayer-Davis EJ Kahkoska AR Jefferies C Dabelea D Balde N Gong CX. Ispad Clinical Practice Consensus Guidelines 2018: Definition, Epidemiology, and Classification of Diabetes in Children and Adolescents. Pediatr Diabetes (2018) 19 Suppl 27(Suppl 27):7–19. doi: 10.1111/pedi.12773 PMC7521365 30226024

[12] Kim JH Yun S Hwang SS Shim JO Chae HW Lee YJ. The 2017 Korean National Growth Charts for Children and Adolescents: Development, Improvement, and Prospects. Korean J Pediatr (2018) 61(5):135–49. doi: 10.3345/kjp.2018.61.5.135 PMC5976563 29853938

[13] Wolfsdorf JI Glaser N Agus M Fritsch M Hanas R Rewers A. Ispad Clinical Practice Consensus Guidelines 2018: Diabetic Ketoacidosis and the Hyperglycemic Hyperosmolar State. Pediatr Diabetes (2018) 19 Suppl 27:155–77. doi: 10.1111/pedi.12701 29900641

[14] Steele C Hagopian WA Gitelman S Masharani U Cavaghan M Rother KI. Insulin Secretion in Type 1 Diabetes. Diabetes (2004) 53(2):426–33. doi: 10.2337/diabetes.53.2.426 14747294

[15] Törn C Landin-Olsson M Lernmark A Palmer JP Arnqvist HJ Blohmé G. Prognostic Factors for the Course of Beta Cell Function in Autoimmune Diabetes. J Clin Endocrinol Metab (2000) 85(12):4619–23. doi: 10.1210/jcem.85.12.7065 11134117

[16] Keenan HA Sun JK Levine J Doria A Aiello LP Eisenbarth G. Residual Insulin Production and Pancreatic Á-Cell Turnover After 50 Years of Diabetes: Joslin Medalist Study. Diabetes (2010) 59(11):2846–53. doi: 10.2337/db10-0676 PMC2963543 20699420

[17] Panero F Novelli G Zucco C Fornengo P Perotto M Segre O. Fasting Plasma C-Peptide and Micro- and Macrovascular Complications in a Large Clinic-Based Cohort of Type 1 Diabetic Patients. Diabetes Care (2009) 32(2):301–5. doi: 10.2337/dc08-1241 PMC2628697 19017769

[18] Sherr JL Ghazi T Wurtz A Rink L Herold KC. Characterization of Residual B Cell Function in Long-Standing Type 1 Diabetes. Diabetes Metab Res Rev (2014) 30(2):154–62. doi: 10.1002/dmrr.2478 24115337

[19] Grönberg A Espes D Carlsson PO. Better Hba1c During the First Years After Diagnosis of Type 1 Diabetes Is Associated With Residual C Peptide 10 Years Later. BMJ Open Diabetes Res Care (2020) 8(1):e00819. doi: 10.1136/bmjdrc-2019-000819 PMC7206906 32107263

[20] Lee TH Kwon AR Kim YJ Chae HW Kim HS Kim DH. The Clinical Measures Associated With C-Peptide Decline in Patients With Type 1 Diabetes Over 15 Years. J Korean Med Sci (2013) 28(9):1340–4. doi: 10.3346/jkms.2013.28.9.1340 PMC3763109 24015040

[21] Luppi P Drain N To R Stolz D Wallace C Watkins S. Autocrine C-Peptide Protects Ins1 B Cells Against Palmitic Acid-Induced Oxidative Stress in Peroxisomes by Inducing Catalase. Endocrinol Diabetes Metab (2020) 3(3):e00147. doi: 10.1002/edm2.147 32704568 PMC7375117

[22] Luppi P Drain P. Autocrine C-Peptide Mechanism Underlying Ins1 Beta Cell Adaptation to Oxidative Stress. Diabetes Metab Res Rev (2014) 30(7):599–609. doi: 10.1002/dmrr.2528 24459093

[23] Cifarelli V Geng X Styche A Lakomy R Trucco M Luppi P. C-Peptide Reduces High-Glucose-Induced Apoptosis of Endothelial Cells and Decreases Nad(P)H-Oxidase Reactive Oxygen Species Generation in Human Aortic Endothelial Cells. Diabetologia (2011) 54(10):2702–12. doi: 10.1007/s00125-011-2251-0 21773684

[24] Thivolet C Marchand L Chikh K. Inappropriate Glucagon and Glp-1 Secretion in Individuals With Long-Standing Type 1 Diabetes: Effects of Residual C-Peptide. Diabetologia (2019) 62(4):593–7. doi: 10.1007/s00125-018-4804-y 30612138

[25] McGee P Steffes M Nowicki M Bayless M Gubitosi-Klug R Cleary P. Insulin Secretion Measured by Stimulated C-Peptide in Long-Established Type 1 Diabetes in the Diabetes Control and Complications Trial (Dcct)/Epidemiology of Diabetes Interventions and Complications (Edic) Cohort: A Pilot Study. Diabetes Med (2014) 31(10):1264–8. doi: 10.1111/dme.12504 PMC4167980 24836354

[26] Lachin JM McGee P Palmer JP. Impact of C-Peptide Preservation on Metabolic and Clinical Outcomes in the Diabetes Control and Complications Trial. Diabetes (2014) 63(2):739–48. doi: 10.2337/db13-0881 PMC3900540 24089509

[27] Lam A Dayan C Herold KC. A Little Help From Residual B Cells Has Long-Lasting Clinical Benefits. J Clin Invest (2021) 131(3):e143683. doi: 10.1172/jci143683 PMC7843219 33529163

[28] Williams KV Becker DJ Orchard TJ Costacou T. Persistent C-Peptide Levels and Microvascular Complications in Childhood Onset Type 1 Diabetes of Long Duration. J Diabetes Complications (2019) 33(9):657–61. doi: 10.1016/j.jdiacomp.2019.05.019 PMC6690760 31239235

[29] Sørensen JS Johannesen J Pociot F Kristensen K Thomsen J Hertel NT. Residual B-Cell Function 3-6 Years After Onset of Type 1 Diabetes Reduces Risk of Severe Hypoglycemia in Children and Adolescents. Diabetes Care (2013) 36(11):3454–9. doi: 10.2337/dc13-0418 PMC3816898 23990516

[30] Rickels MR Evans-Molina C Bahnson HT Ylescupidez A Nadeau KJ Hao W. High Residual C-Peptide Likely Contributes to Glycemic Control in Type 1 Diabetes. J Clin Invest (2020) 130(4):1850–62. doi: 10.1172/jci134057 PMC7108933 31895699

[31] Babaya N Noso S Hiromine Y Taketomo Y Niwano F Yoshida S. Relationship of Continuous Glucose Monitoring-Related Metrics With Hba1c and Residual B-Cell Function in Japanese Patients With Type 1 Diabetes. Sci Rep (2021) 11(1):4006. doi: 10.1038/s41598-021-83599-x 33597626 PMC7889608

[32] Hao W Gitelman S DiMeglio LA Boulware D Greenbaum CJ. Fall in C-Peptide During First 4 Years From Diagnosis of Type 1 Diabetes: Variable Relation to Age, Hba1c, and Insulin Dose. Diabetes Care (2016) 39(10):1664–70. doi: 10.2337/dc16-0360 PMC5033079 27422577

[33] Samuelsson U Lindblad B Carlsson A Forsander G Ivarsson S Kockum I. Residual Beta Cell Function at Diagnosis of Type 1 Diabetes in Children and Adolescents Varies With Gender and Season. Diabetes Metab Res Rev (2013) 29(1):85–9. doi: 10.1002/dmrr.2365 PMC3644881 23081842

[34] Miao H Zhang J Gu B Gao A Hong J Zhang Y. Prognosis for Residual Islet B-Cell Secretion Function in Young Patients With Newly Diagnosed Type 1 Diabetes. J Diabetes (2019) 11(10):818–25. doi: 10.1111/1753-0407.12912 30848017

[35] Barker A Lauria A Schloot N Hosszufalusi N Ludvigsson J Mathieu C. Age-Dependent Decline of B-Cell Function in Type 1 Diabetes After Diagnosis: A Multi-Centre Longitudinal Study. Diabetes Obes Metab (2014) 16(3):262–7. doi: 10.1111/dom.12216 24118704

[36] Szypowska A Groele L Wysocka-Mincewicz M Mazur A Lisowicz L Ben-Skowronek I. Factors Associated With Preservation of C-Peptide Levels at the Diagnosis of Type 1 Diabetes. J Diabetes Complications (2018) 32(6):570–4. doi: 10.1016/j.jdiacomp.2018.03.009 29699766

[37] Ludvigsson J Carlsson A Deli A Forsander G Ivarsson SA Kockum I. Decline of C-Peptide During the First Year After Diagnosis of Type 1 Diabetes in Children and Adolescents. Diabetes Res Clin Pract (2013) 100(2):203–9. doi: 10.1016/j.diabres.2013.03.003 23529064

[38] Lauria A Barker A Schloot N Hosszufalusi N Ludvigsson J Mathieu C. Bmi Is an Important Driver of B-Cell Loss in Type 1 Diabetes Upon Diagnosis in 10 to 18-Year-Old Children. Eur J Endocrinol (2015) 172(2):107–13. doi: 10.1530/eje-14-0522 25378371

[39] Effect of Intensive Therapy on Residual Beta-Cell Function in Patients With Type 1 Diabetes in the Diabetes Control and Complications Trial. A Randomized, Controlled Trial. The Diabetes Control and Complications Trial Research Group. Ann Intern Med (1998) 128(7):517–23. doi: 10.7326/0003-4819-128-7-199804010-00001 9518395

