# Supplementary material for: Insulin Requirement and Complications Associated With Serum C-Peptide Decline in Patients With Type 1 Diabetes Mellitus During 15 Years After Diagnosis
Source: Front Endocrinol (Lausanne). 2022 Apr 19;13:869204. doi: 10.3389/fendo.2022.869204 (PMC9061978; doi:10.3389/fendo.2022.869204)
Supplement: Supplementary Table 1 — Presence of diabetes complications at each time point. Data are presented as number of patients (%). Diabetic ketoacidosis event at initial diagnosis is excluded. [file Table_1.docx]

**Supplementary Table 1.** Presence of diabetes complications at each time point

|  | **Baseline** | **Year 1** | **Year 3** | **Year 5** | **Year 10** | **Year 15** |
| --- | --- | --- | --- | --- | --- | --- |
| Diabetic retinopathy | 0 (0.0%) | 0 (0.0%) | 1 (0.4%) | 5 (2.1%) | 23 (9.8%) | 60 (25.6%) |
| Peripheral neuropathy | 0 (0.0%) | 0 (0.0%) | 2 (0.9%) | 10 (4.3%) | 26 (11.1%) | 54 (23.1%) |
| Diabetic nephropathy | 0 (0.0%) | 2 (0.9%) | 5 (2.1%) | 12 (5.1%) | 28 (12.0%) | 52 (22.2%) |
| Diabetic ketoacidosis | 0 (0.0%) | 19 (8.1%) | 24 (10.3%) | 32 (13.7%) | 37 (15.8%) | 39 (16.7%) |

*Data are presented as number of patients (%).*

*Diabetic ketoacidosis event at initial diagnosis is excluded.*
